# Relationship between Pain and Medial Meniscal Extrusion in Knee Osteoarthritis

**DOI:** 10.1155/2015/210972

**Published:** 2015-12-15

**Authors:** Hiroaki Kijima, Shin Yamada, Koji Nozaka, Hidetomo Saito, Yoichi Shimada

**Affiliations:** Department of Orthopedic Surgery, Akita University Graduate School of Medicine, 1-1-1 Hondo, Akita 010-8543, Japan

## Abstract

*Purpose*. In knee osteoarthritis, the degree of pain varies despite similar imaging findings. If there were quantitative findings related to the pain of knee osteoarthritis, it could be used for diagnosis or screening. The medial meniscal extrusion was investigated as a candidate quantitative finding related to the pain of knee osteoarthritis.* Methods*. Seventy-six knees of 38 patients (mean age, 73 years) who received intra-articular injections of hyaluronic acid into unilateral knees at the time of diagnosis of knee arthritis were investigated. Cartilage thickness of the femoral medial condyle and medial meniscal extrusion of bilateral knees were measured by ultrasonography. Thirty-eight knees that had hyaluronic acid injections were compared with 38 other side knees from the same patients as the control group.* Results*. The average cartilage thicknesses of the knees with pain that received intra-articular injections and the knees without pain that received no injections were 1.02 and 1.05 mm, respectively (*P* = 0.6394). On the other hand, the average medial meniscal extrusions of the knees with and without pain were 7.58 and 5.88 mm, respectively (*P* = 0.0005); pain was associated with greater medial meniscal extrusions.* Conclusion*. Medial meniscal extrusion is a quantitative finding related to the pain of knee osteoarthritis.

## 1. Introduction

There are patients who have knees with no pain that show findings of osteoarthritis on X-ray images or magnetic resonance images (MRI). In other words, asymptomatic osteoarthritis of the knee exists, and asymptomatic knee osteoarthritis rarely becomes the target of treatment.

In recent years, large-scale epidemiological investigations of knee osteoarthritis have been carried out [1–4]. The diagnosis of knee osteoarthritis in these investigations has been made based on spur formation and joint space narrowing on X-ray images or cartilage degeneration on MRI. However, these imaging findings are not related to pain, which is the target of treatment. The results of epidemiological investigations using the above method differ from those of symptomatic knee osteoarthritis targeted for treatment, because the results include a considerable number of asymptomatic knee osteoarthritis cases.

It is thus useful to investigate the epidemiology of symptomatic knee osteoarthritis to standardize the treatment policy for knee osteoarthritis. However, an index related to the pain of knee osteoarthritis is necessary to investigate the epidemiology of symptomatic knee osteoarthritis. If there were a quantitative imaging finding related to the degree of pain, it could be used in the diagnostic criteria for knee osteoarthritis or for screening for knee osteoarthritis. Therefore, we hypothesized that medial meniscal extrusion (MME) in knee osteoarthritis is a candidate quantitative imaging finding related to the degree of pain.

MME occurs when the medial meniscus is displaced medially and extrudes from the joint. Kenny was the first to report MME and found that radial displacement of the medial meniscus may be related to a loss of meniscal function [5]. After the first report, many studies reported that MME is related to the progress of knee osteoarthritis. Furthermore, it was found that MME reflects cartilage damage more clearly than X-ray findings [6].

On the other hand, pain in the medial joint space during weight-bearing is a typical symptom of knee osteoarthritis, but the cause of this pain remains unclear. However, it has been confirmed that MME is greater during weight-bearing than during non-weight-bearing [7]. This phenomenon may cause tension of the tissue around the medial joint space and lead to the pain in knee osteoarthritis through mechanoreceptors. Therefore, the purpose of this study was to clarify the relationship between pain and MME in knee osteoarthritis.

## 2. Materials and Methods

A total of 76 knees of 38 patients (22 males, 54 females; average age, 73 years; age range, 49–89 years) who presented with unilateral knee pain and who received intra-articular injections of hyaluronic acid into unilateral knees at the time of diagnosis of knee osteoarthritis were studied. Informed consent was obtained from all subjects, and institutional review board approval for this study was obtained from the Department of Orthopedic Surgery, Ugo Municipal Hospital, in which all subjects were treated. The chief complaint of all cases was pain at the medial aspect of the knee, and the grade of osteoarthritis on X-ray findings of all cases was Kellgren-Lawrence grade 2, 3, or 4.

**Figure 1:**
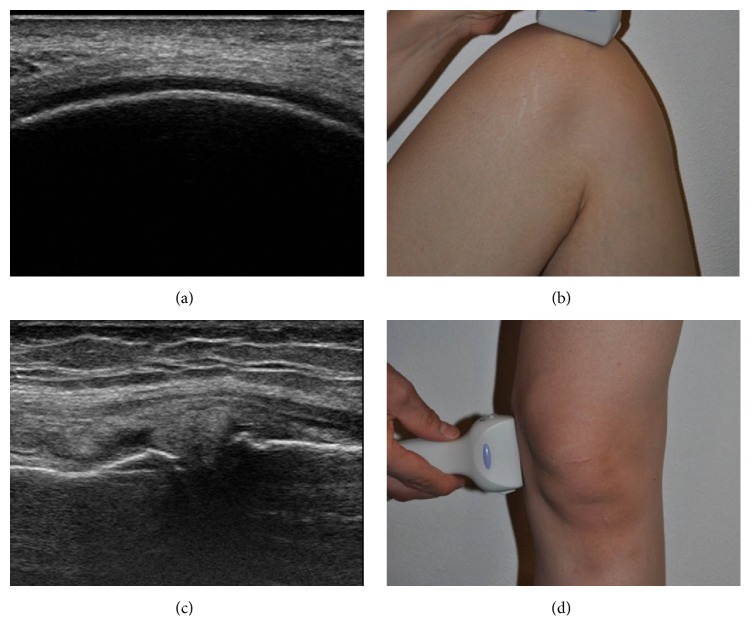
(a) Cartilage thickness of the femoral medial condyle. (b) Depiction of the femoral cartilage by putting the probe on the weight-bearing surface. (c) Radial displacement of the medial meniscus. (d) Depiction of radial displacement by putting the probe on the medial joint space.

The cartilage of each femoral medial condyle (weight-bearing region) (Figure 1(a)) was first depicted by putting the ultrasound probe (ProSound *α*7, Hitachi Aloka Medical, Tokyo, Japan) on the femoral medial condyle (weight-bearing region) with the knee flexed (Figure 1(b)). The thickness of the cartilage was measured at the femoral medial condyle (weight-bearing region) using the method of Saarakkala et al. [8].

Next, the MME (Figure 1(c)) of each knee was depicted by putting the ultrasound probe on the medial joint space with the knee extended (Figure 1(d)). The amount of MME was measured using the method of Kawaguchi et al. [7].

The knees that received joint injections were defined as the knees with pain, and the knees that did not receive joint injections were defined as the knees without pain. The amount of MME and the thickness of the cartilage were compared between the two groups using Student's *t*-test. Significance was set at the *P* < 0.05 level.

## 3. Results

**Figure 2:**
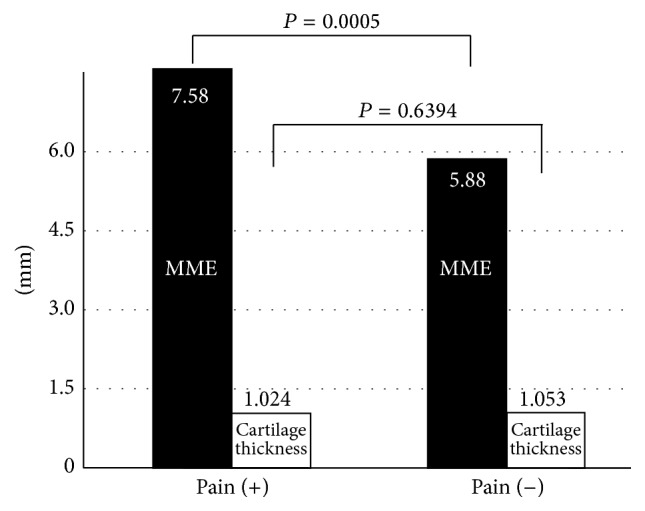
Relationship between pain and medial meniscal extrusion or the cartilage thickness of the femoral medial condyle. The average cartilage thicknesses of the knees with and without pain were 1.02 and 1.05 mm, respectively, with no significant difference (*P* = 0.6394). The average medial meniscal extrusions (MMEs) of the knees with and without pain were 7.58 and 5.88 mm, respectively; knees with pain had greater MMEs than knees without pain (*P* = 0.0005).

The average thickness of the cartilage at the femoral medial condyle (weight-bearing region) of the knees with pain was 1.02 ± 0.28 mm and that of the knees without pain was 1.05 ± 0.26 mm; no significant difference was observed (*P* = 0.6394) (Figure 2). In other words, the degree of progress of the osteoarthritis was similar between the knees with and without pain. On the other hand, the average MME of the knees with pain was 7.58 ± 2.16 mm, while that of the knees without pain was 5.88 ± 1.90 mm. The knees with pain had significantly greater MME than those without pain (*P* = 0.0005) (Figure 2).

## 4. Discussion

In knees with cartilage (weight-bearing region) with the same amount of thinning, MME was significantly greater in knees with pain than in those without pain. In other words, it appears that the amount of MME could become an index related to the pain of knee osteoarthritis.

The reliability of cartilage evaluation with ultrasonography has already been reported, and it has been shown that cartilage evaluation with ultrasonography is related to cartilage evaluation on arthroscopy [8]. On the other hand, there have been numerous reports concerning the evaluation of MME of the knees with ultrasonography in recent years. MME of the knees measured by ultrasonography has been shown to be related to the progress of knee osteoarthritis, similar to that of MME of the knees measured by MRI [7]. By using the two methods mentioned above, the present study demonstrated that MME was related to the pain when the degree of the pain was different, even though cartilage thickness, which is the gold standard for conventional knee osteoarthritis evaluation, was the same.

It is well known the medial meniscal complete radial tear or root tear results in meniscal extrusion and pain out of proportion to a typical medial meniscus tear or osteoarthritis. Conversely, in the knees whose meniscus remains intact, there is less pain. Therefore, MME measured in this study may be simply making the diagnosis of a complete radial tear or root tear of the medial meniscus.

However, because MME was evaluated with ultrasonography and not MRI, it was not only noninvasive but also very easy and low-cost evaluation. Other reports have shown that MME measured by ultrasonography is useful for medical examinations of the general population [9]. In addition, it has been shown that the method to measure MME by ultrasonography has high reliability [10].

One of the limitations of this study is that it involved knees of patients attending an orthopedic outpatient department, whose osteoarthritis grade was greater than Kellgren-Lawrence grade 2. In other words, we cannot know when MME starts to be related to the pain in knee osteoarthritis, because no cases of early knee arthritis were included in the present study. In addition, the results of this study cannot be used to consider the effects of surgery to decrease MME at the early stage of knee osteoarthritis (e.g., suturing of the medial meniscus) on the pain of knee osteoarthritis.

Another limitation of this study is the small number of knees. A larger scale investigation of MME involving the general population is needed.

The pain at the medial joint space during weight-bearing, which is the typical symptom of knee osteoarthritis, may occur because the soft tissue around the medial joint space is under tension during weight-bearing. This is because the MME increases during weight-bearing compared to non-weight-bearing [7]. However, a medial meniscal complete radial tear or a root tear [11, 12], which is considered to be a cause of MME in itself, may be the cause of the pain in knee osteoarthritis, so the cause of the pain cannot be explained in the present study. In other words, because a medial meniscal complete radial tear or a root tear results in biomechanical loads equal to a complete medial meniscectomy, the pain from MME may not be the pain of osteoarthritis, but the pain of losing the function of the meniscus.

On the other hand, it became clear from the results of this study that the amount of MME, which could be investigated noninvasively in several seconds with ultrasonography, was related to the pain in knee osteoarthritis. Therefore, if this method is used, it is easy to perform a large-scale epidemiological investigation of symptomatic knee osteoarthritis, which is the target of treatment, rather than conventional knee osteoarthritis diagnosed by imaging alone.

In other words, the results of this study may be related to the discovery of new evidence of knee osteoarthritis, which is related to the standardization of treatment and the establishment of true diagnostic criteria for knee osteoarthritis.

In addition, the results of this study suggest a mechanism for the pain of knee osteoarthritis that may help develop new treatment methods.

## 5. Conclusions

In knees with the same degree of osteoarthritis, MME in the knee was greater in patients experiencing pain than in those without pain.

## References

[1] Yoshimura N., Muraki S., Oka H., Kawaguchi H., Nakamura K., Akune T. (2011). Association of knee osteoarthritis with the accumulation of metabolic risk factors such as overweight, hypertension, dyslipidemia, and impaired glucose tolerance in Japanese men and women: the ROAD study. *Journal of Rheumatology*.

[2] Yoshimura N., Muraki S., Oka H., Kawaguchi H., Nakamura K., Akune T. (2010). Cohort profile: research on osteoarthritis/osteoporosis against disability (ROAD) study. *International Journal of Epidemiology*.

[3] Muraki S., Oka H., Akune T. (2009). Prevalence of radiographic knee osteoarthritis and its association with knee pain in the elderly of Japanese population-based cohorts: the ROAD study. *Osteoarthritis and Cartilage*.

[4] Oka H., Muraki S., Akune T. (2008). Fully automatic quantification of knee osteoarthritis severity on plain radiographs. *Osteoarthritis and Cartilage*.

[5] Kenny C. (1997). Radial displacement of the medial meniscus and Fairbank's signs. *Clinical Orthopaedics and Related Research*.

[6] Ohi G., Kimura M., Asagumo H., Kanbayashi S., Kobayashi A., Taki M. (2006). The relation between radial displacement of medial meniscus and grade of chondral lesion in early osteoarthritis of the knee. *East Japan Journal of Orthopaedic and Traumatology*.

[7] Kawaguchi K., Enokida M., Otsuki R., Teshima R. (2012). Ultrasonographic evaluation of medial radial displacement of the medial meniscus in knee osteoarthritis. *Arthritis & Rheumatism*.

[8] Saarakkala S., Waris P., Waris V. (2012). Diagnostic performance of knee ultrasonography for detecting degenerative changes of articular cartilage. *Osteoarthritis and Cartilage*.

[9] Yanagisawa S., Ohsawa T., Saito K., Kobayashi T., Yamamoto A., Takagishi K. (2014). Morphological evaluation and diagnosis of medial type osteoarthritis of the knee using ultrasound. *Journal of Orthopaedic Science*.

[10] Aki T., Takahashi A., Kashiwaba M., Yamamoto N., Kamimura M., Itoi E. (2013). Quantitative evaluation for the medial meniscal extrusion using ultrasonography. *Tohoku Journal of Orthopaedics and Traumatology*.

[11] Pagnani M. J., Cooper D. E., Warren R. F. (1991). Extrusion of the medial meniscus. *Arthroscopy*.

[12] Bin S.-I., Kim J.-M., Shin S.-J. (2004). Radial tears of the posterior horn of the medial meniscus. *Arthroscopy*.

